# SSPRD: A Shared-Storage-Based Hardware Packet Reordering and Deduplication System for Multipath Transmission in Wide Area Networks

**DOI:** 10.3390/mi15111323

**Published:** 2024-10-30

**Authors:** Jiandong Ma, Zhichuan Guo, Mangu Song

**Affiliations:** 1 National Network New Media Engineering Research Center, Institute of Acoustics, Chinese Academy of Sciences, No. 21, North Fourth Ring Road, Haidian District, Beijing 100190, China; majd@dsp.ac.cn (J.M.); songmg@dsp.ac.cn (M.S.); 2 School of Electronic, Electrical and Communication Engineering, University of Chinese Academy of Sciences, No. 19(A), Yuquan Road, Shijingshan District, Beijing 100049, China; 3 Suzhou Haiwang Network Technologies Co., Ltd., Suzhou 215163, China

**Keywords:** packet reordering, multipath transmission, WAN, FPGA

## Abstract

To increase bandwidth and overcome packet loss in Wide Area Networks (WANs), per-packet multipath transmission and redundant transmission are increasingly being used as Software-Defined Wide Area Network (SD-WAN) solutions. However, this results in out-of-order and duplicate packets in the destination network. To restore sequential and unique data streams for multiple connections, hardware packet buffers with significant depth are required due to the large delay difference between WAN paths. To address this issue, SSPRD, a shared-storage-based packet reordering and deduplication system using a Field-Programmable Gate Array (FPGA), is proposed. The storage space for packets and sub-buffers is shared by all sessions with dynamic allocation. Packets are stored in the DDR and are sorted by their descriptors in the buffers. We also develop a sub-buffer-based timeout event handling algorithm. While supporting four sessions, SSPRD achieves a deep reorder buffer on hardware, with a depth of up to 15,360 packets per session. Compared with other solutions, SSPRD reduces buffer space usage by 62.5%, and reaches a packet reordering and deduplicating performance of 10 Gbps for 1500-byte packets.

## 1. Introduction

Wide Area Networks (WANs) are telecommunications networks that extend over a large geographical area, including enterprise networks, education networks, and the Internet. Users can access WANs through various wired or wireless methods provided by different Internet Service Providers (ISPs).

However, compared to Local Area Networks (LANs), WANs, especially the Internet, suffer from low bandwidth, high latency, and occasional packet loss and disorder [1]. For enterprises which have branch offices located in a separate geographic area, the limited and unstable performance of the Internet leads to problems such as application response timeouts, slow file transfers, and video lag in online meetings between branch offices, between branch offices and headquarters, or between data centers.

To address this issue, WAN optimization [2] based on Software-Defined Wide Area Network (SD-WAN) architecture is proposed. Multipath transmission and redundant transmission are two typical optimization methods. In the SD-WAN solution of Huawei [3], “multi-send-selective-receive” transmission (redundant transmission) and “per-packet load balancing” transmission are proposed. Multipath transmission allows data streams to be transmitted via different paths to increase bandwidth [4] and load balancing. Per-packet multipath transmission, capable of splitting “elephant flows” [5], outperforms traditional per-flow multipath transmission and is widely deployed in new network architectures, such as Information-Centric Networking (ICN) [6], and other network environments such as Data Center Networks (DCNs) [7] and mobile networks [8]. Redundant transmission replicates data packets and transmits them simultaneously through different paths to fix packet loss and reduce latency [9].

However, multipath transmission and redundant transmission lead to packet disorder and duplication at the destination network. Unfortunately, most applications and transport protocols do not support handling a large number of out-of-order or duplicate packets. QUIC, TCP, RoCEv2, and other transport protocols with data integrity checks assume that packet loss occurs when receiving disordered packets, and trigger retransmission or congestion window reduction, severely impacting performance. Moreover, sorting packets or discarding duplicate packets through a network stack or user software will consume significant CPU cycles and PCIe-Memory bandwidth at the destination host, which affects other applications. The Customer Premises Equipment (CPE) devices [3] at the destination network achieve packet reordering and deduplicating. However, the implementation details are not disclosed.

Therefore, a hardware packet reordering and deduplication system is required for the Customer Premises Equipment (CPE) located in the destination SD-WAN network when applying multipath transmission and redundant transmission. The packets can be identified by packet number (PN). The system can also be used for PN-based network protocol packet sorting and deduplicating, such as RTP, QUIC, RoCEv2, and other user-defined protocols. The system should be able to deduplicate and reorder the incoming packet streams and forward the processed streams to the network hosts that are running multiple kinds of user applications.

For reordering, the system should have a packet buffer whose depth is greater than the Out-of-Order Degree [10], as well as the Maximum Packet Disorder Distance, max_PDD, for each session. In this paper, network delay refers to the time it takes for a packet to travel from the source to the destination over a network, which is the one-trip time or half of the Round-Trip Time (RTT). Because multipath transmission is a reason for packet disordering, max_PDD is mainly influenced by the Maximum Multipath Delay Difference, max_MDD. We investigated the value of max_MDD in WANs. According to network measurements performed by Xu [11] in China, from Chongqing to Gansu (which are separated by a distance of 985km), the backbone delay varies from about 7.5 ms to nearly 27.5 ms due to different routing paths. PingPlotter [12] suggested that network latency is related to distance and connection type. Every 120 miles causes 1 ms of delay, and the inherent delay of cable internet is between 2.5 ms and 20 ms. Tianxia [13] compares the latency between a public network and an SD-WAN optimized network. Due to the use of different networks, most of the delay differences are within 7.5 ms, with the maximum being 16.25 ms (Hong Kong–Frankfurt). Considering the measurement results above and delay jitter [14], we assume that the maximum delay difference between multiple WAN paths does not exceed 17.5 ms.

At a *B* bandwidth with an MTU packet size, the Maximum Packet Disorder Distance max_PDD at the receiver can be calculated by max_MDD using Equation (1).

(1) $$max\_PDD=\frac{B\times max\_MDD}{MTU}.$$

For WANs, the typical bandwidth is 10 Gbps. And in this study, we aimed to support 1500-byte packets, which is the Maximum Transmission Unit (MTU) in WANs. When B=10Gbps, max_MDD=17.5ms, and MTU=1500B, we have max_PDD = 14,583.

Therefore, in order to handle out-of-order packets in an environment with a 10 Gbps bandwidth, a 1500-byte packet size, and a 17.5 ms max multipath delay difference, the packet reordering and deduplication system should have a buffer with a depth of 14,583 packets per session while supporting multiple sessions.

Current hardware packet reordering and deduplication methods mainly use data structures such as chains (linked lists) [15], Content-Addressable Memory (CAM), shift registers [16], Random Access Memory (RAM) (ring buffer, array) [17,18,19,20], and parallel FIFO (First-In First-Out) memories. They are suitable for specific disorder modes, assuming rare pkt loss or a small max_PDD. When the depth of the buffer increases, these methods require large logic or storage resources. Alternatively, a packet-metadata-based sorting approach [21] has been proposed, enabling the sorting of 512 packets. However, as the max_PDD and the number of connections increase, the module occupies too many logical and storage resources to be implemented on a chip.

To address this issue, we propose SSPRD, a Shared-Storage-based hardware Packet Reordering and Deduplication system. We sort the packets by their descriptors instead of themselves. For the incoming packets which need to be cached, we store them in the DDR and place their packet descriptors in the session buffers for reordering. Each session’s buffer consists of 16 sub-buffers and there are 1024 packet descriptors in each sub-buffer. The main contributions of this work are as follows:

- The storage space of packets and packet descriptor sub-buffers is shared by all sessions with dynamic allocation. Due to the shared storage strategy and packet timeout mechanism, their space usage remains generally constant regardless of the session count.
- A sub-buffer-based timeout event handling algorithm is proposed. We create a timeout event only for the first packet in a sub-buffer. If the first packet in a sub-buffer has timed out, we output the timeout packets, keep the non-timeout packets in the sub-buffer, and create a new timeout event for the sub-buffer.
- While supporting four sessions, SSPRD achieves deep reorder buffering on hardware, with a depth of up to 15,360 packets per session. Compared with other solutions, SSPRD reduces buffer space usage by 62.5%, and reaches a packet reordering and deduplicating performance of 10 Gbps for 1500-byte packets.

## 2. Related Work

Implementing packet reordering and deduplication on hardware platforms, such as Field-Programmable Gate Array (FPGA) or Network Interface Card (NIC), not only leverages the benefits of its high performance and low power consumption, but also minimizes the usage of CPU and PCIe bandwidth for the receiver hosts.

TCP/IP Offload Engine (TOE) is a hardware component that aims to offload TCP protocol processing from the CPU. TCP reassembly, involving packet reordering, is one of its key functions. Zhou [17] proposes a packet reordering scheme on FPGA, supporting a sorting depth of 43 discontinuous TCP packets. It uses two separate RAMs to store the start and end sequence numbers and the DDR storage addresses of out-of-order packets. A scanning method is established to sort and merge frames, similar to that used in [22,23]. Although it supports a number of sessions, it is only suitable for specific out-of-order patterns caused by packet loss or disorder with low probability. If most incoming packets are out of order with a large disorder distance, the sorting buffer will be quickly exhausted.

Network Processors (NPs) commonly leverage multiple CPU cores to enhance packet processing throughput, often introducing packet disorder as a consequence. Meitinger [18] employs a circular buffer for sorting and discusses the sequence number wrap-around issue. Wu [15] establishes a chain-based sorting structure, and Traboulsi [20] enhances the management capability of circular queues. However, these methods assume no packet loss, and related data structures do not support much deeper sorting. Song et al. [24] enable in-network packet reordering in Data Center Networks with the help of P4 programmable switches. which is not feasible in WANs.

Hoang [16] proposes a sorting circuit based on shift registers that is able to insert an out-of-order packet in the correct order in two clock cycles. However, the circuit is too complex to achieve a deeper list. For a 16-bit sequence number, a 512-depth packet sorting list consumes 80% of the Adjustable Logic Modules (ALMs) of a Cyclone V FPGA board.

To improve the sorting depth, Ukon [19] uses a packet array with 480 members for each session, and each member can store a packet no larger than 2KB. However, storing packets and buffers on a chip would occupy too many resources in the scenario of our study. MELO [25] implements a linked list of bitmap blocks to achieve selective loss recovery transport and efficient memory management, but the proposed linked list does not support random access. LEFT [26] improves MELO’s performance with a recently used cache. However, a cache is only a local optimization method for performance, and introduces additional design complexity; the random access performance of the linked list is still unstable. Additionally, in [18,25,26], the systems are simulated but not implemented on hardware.

For higher memory efficiency, Beneš [21] proposes a packet reordering unit based on packet metadata. After listing the shortcomings of packet arrays, parallel FIFOs, and other methods of resource consumption, the author chooses to store the out-of-order packets in DRAM and sorts their metadatas on a chip using an array. The metadata consist of the sequence number, the DRAM address, and the packet length. Although the use of metadata reduces buffer space usage, there is no theoretical support for how much space should be occupied by packet storage without packet timeout management, introducing the risk of space exhaustion during packet loss. Moreover, this method has poor scalability and performance. The space usage of the buffers increases linearly with the number of sessions, and the design stores all the incoming packets in the DDR regardless of their sequence number, which leads to a bottleneck in the system throughput.

## 3. System Design

### 3.1. Design Overview

**Figure 1:**
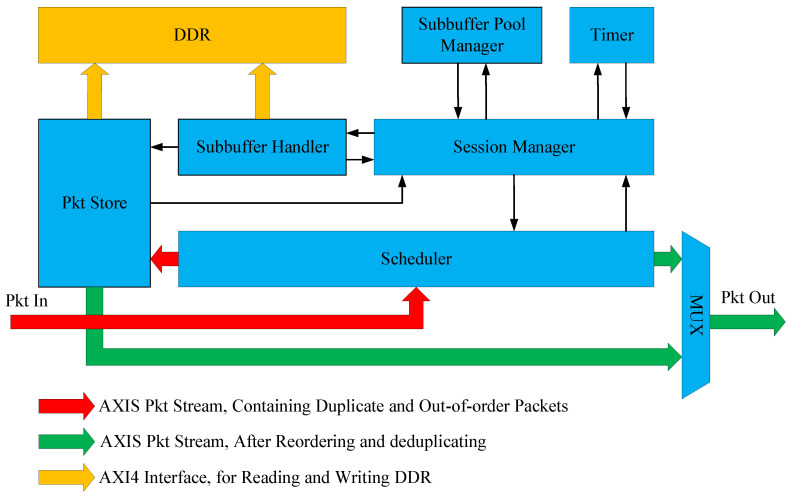
System overview of SSPRD.

Based on the packet buffer in the DDR, SSPRD transforms redundant and out-of-order data streams into ordered and unique data streams. The architecture of SSPRD is illustrated in Figure 1. The red arrows represent the AXIS packet stream containing duplicate and out-of-order packets, entering the system from the left in the diagram. The green arrows represent the AXIS packet stream sorted and deduplicated by the system, exiting from the right in the diagram. The yellow arrows denote the AXI4 data interface to the DDR, used for reading and writing packets and sub-buffers. The black arrows indicate information channels between modules.

The system is composed of multiple modules. The Session Manager serves as the primary logic processing module, handling packet and timeout events, and maintaining the Expected Packet Numbers (EPNs), packet timeout thresholds, and sub-buffer node arrays of sessions. The Scheduler manages packet reception, triggers packet processing requests, and, based on the processing results, either drops packets, caches them in the DDR, or sends them forward. The Timer module handles the creation and triggering of timeout events. The Sub-buffer Pool Manager handles the allocation and release of sub-buffers. The Pkt Store module can store packets in the DDR and output their packet descriptors, or retrieve packets from the DDR based on their packet descriptors. The Sub-buffer Handler module manages the scanning and updating of sub-buffers, which consist of thousands of packet descriptor nodes. The MUX module merges two AXIS packet streams into one output stream.

In the following sections, we describe the idea of our design and the details of each module.

### 3.2. Shared Packet Storage

**Figure 2:**
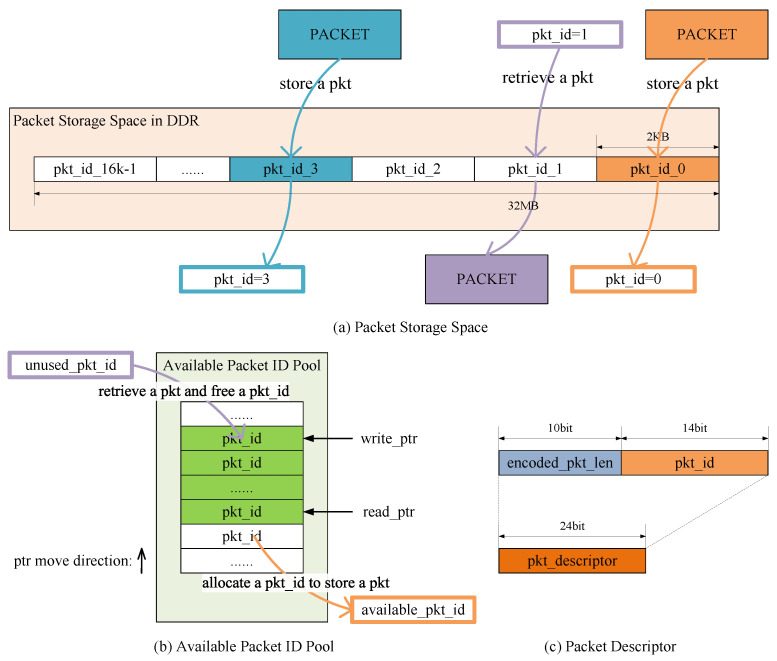
Design of shared packet storage. (**a**) Partition of Packet Storage Space. (**b**) Allocation and freeing of packet IDs in Available Packet ID Pool. (**c**) Content of a packet descriptor.

SSPRD implements shared packet storage, which stores all the packets together and is shared by all sessions. Packets are stored in an area called Packet Storage Space, as shown in Figure 2a, and identified by packet descriptors. A Packet descriptor, as shown in Figure 2c, consists of packet length and packet ID. The blocks of the Packet Storage Space for storing packets are indexed by packet IDs, which are managed in an area called Available Packet ID Pool, as shown in Figure 2b. The Pkt Store manages the Packet Storage Space and the Available Packet ID Pool. It can store the incoming packets in the DDR and output their descriptors, or retrieve the packets from the DDR according to their descriptors. The separation of packet storage and reordering functions allows packets to be sorted by their descriptors instead of themselves in the buffer, which greatly reduces the space usage of buffers.

Next, we determine a reasonable size for the Pkt Storage Space. Because of the shared packet storage strategy, after the packets are reordered and output by the system, their space can be reused by other packets. Therefore, the size of the Packet Storage Space is related to the completion time of reordering. We use the term “packet hole” to describe a gap where packets have not arrived yet in the reorder buffer. Assuming the maximum multipath delay difference is 17.5 ms and there is no packet loss, any packet hole can be filled within 17.5 ms. Since there are 14,583 incoming packets in 17.5 ms under a 10 Gbps bandwidth and a 1500-byte packet size, as calculated before, a 16,384-packet-depth Packet Storage Space will never be exhausted without packet loss.

However, even with multipath and redundant transmission, packet loss in WANs cannot be avoided completely, ultimately causing long-lived packet holes and exhaustion of the Packet Storage Space. Hence, we developed a packet timeout mechanism in the buffer. The packet timeout threshold of a session is configured by the user based on applications or network conditions, and its maximum is 17.5 ms as calculated before. The system evicts timeout packets from the buffer and outputs them.

In addition, SSPRD is responsible for packet sorting and deduplication. In cases of packet loss, SSPRD outputs the packet stream directly to the receiver of the network. User applications can handle packet loss in various ways based on their service type. On the one hand, applications tolerant of packet loss (e.g., live video streaming, voice chatting) can use the ordered and deduplicated data stream directly. On the other hand, applications that check data integrity (e.g., large file transfers) can easily trigger retransmission based on the continuity of the data stream PN, thereby reducing the complexity of the network stack at the receiver.

In summary, with the packet timeout mechanism, a 16,384-packet-depth Packet Storage Space is sufficient for our design. The size of the Packet Storage Space is dependent on the network bandwidth, packet size, and maximum multipath delay differences, independent of the number of sessions.

The Packet Storage Space in the DDR is organized into 16,384 blocks, each 2 KB in size and indexed by packet ID, as depicted in Figure 2a. Each block has enough space to store an entire packet because the MTU for WANs is commonly 1500 bytes. The proposed design of Packet Storage Space is free of fragmentation issues and complex management due to fixed-size memory allocation. The space occupied by Packet Storage Space is 16,384×2KB=32MB.

To maximize memory utilization, the blocks in the Packet Storage Space are dynamically occupied and released. Each block is identified by its packet ID. The Available Packet ID Pool, maintained as a circular array with read and write pointers (similar to FIFO), stores the idle packet IDs as shown in Figure 2b. Initially, all packet IDs are written into the pool. When a packet needs to be stored in the DDR, the packet ID identified by the read pointer is popped from the pool, indicating the location where the packet should be placed, and the read pointer is incremented by 1. Conversely, when a packet is retrieved from the DDR, its packet ID is written to the position identified by the write pointer, and the write pointer is incremented by 1.

The Available Packet ID Pool is stored on a chip for performance. Because there are 16,384 packet IDs, the width of a packet ID is 14 bits. The Xilinx BRAM36k is a memory block with a width of 72 bits and a depth of 512. Thus, the Available Packet ID Pool occupies 16,384×14bits=224Kbits, that is, 7 BRAMs.

Packets stored in the DDR are identified by packet descriptors, as shown in Figure 2c. A 24-bit packet descriptor consists of a packet ID and an encoded packet length. Additional information, including the precise packet length, can be stored within the packet ID blocks in the DDR.

### 3.3. Shared Buffer Storage

We implemented shared buffer storage for memory efficiency. Packet descriptors are stored and sorted in the buffers of each session, but not every session’s buffer is in use simultaneously. Therefore, in the same way, we designed the Sub-buffer Storage Space and the Available Sub-buffer ID Pool, managed by the Sub-buffer Pool Manager. Considering the logical resource footprint, each session’s buffer consists of 16 sub-buffers, each capable of caching 1024 packet descriptor nodes. Sub-buffers are not reserved for any session, but are shared by all sessions. When a sub-buffer is needed by a session, it will be allocated from the Available Sub-buffer ID Pool. When a sub-buffer is empty, it will be cleared and released.

**Figure 3:**
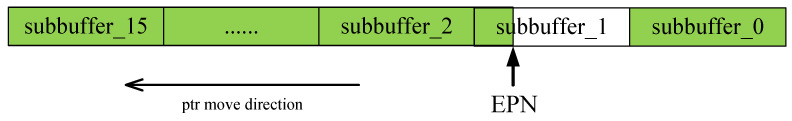
Available part of reorder buffer per session.

The session sub-buffers are indexed sequentially from 0 to 15. A header pointer points to the position of the EPN (Expected Packet Number) in a session buffer. It can move forward with the arrival of sequential packets. The header pointer may point to the middle of a sub-buffer, as shown in Figure 3. The green part of the buffer is the available reorder cache. We choose not to reuse the blank part of the buffer due to design complexity. Therefore, it is conservative to state that, for SSPRD, the depth of the reorder buffer per session is 1024×15=15,360.

Next, we determined the appropriate size for the Sub-buffer Storage Space. The upper-layer SD-WAN application dynamically controls how the 10 Gbps bandwidth is distributed across different sessions. Therefore, the share of the bandwidth decreases the max_PDD of each session. Thus, according to Equation (1), if there is one active stream at a rate of 10 Gbps, a 15,360-depth buffer (which consumes 16 sub-buffers) is enough for reordering. If there are two active streams, each at a rate of 5 Gbps, for example, two 7680-depth buffers are enough for reordering. As the number of sessions increases, the bandwidth of 10 Gbps is shared. Because out-of-order packets can be cached near the boundaries of sub-buffers during transmission, each active session needs at least two sub-buffers. Therefore, the number of sub-buffers required in the Sub-buffer Storage Space is 16+2×Session_Count, where Session_Count is the number of sessions. Additionally, if the session count is 1, the number of sub-buffers needed is 16, because one session could occupy 16 sub-buffers at most.

In the example implementation presented in this paper, the session count is 4 (equal to the session count in Benevs [21]), so there are 16+2×4=24 sub-buffers in the Sub-buffer Storage Space. Hence, the width of a sub-buffer ID is 5 bits. The width of a packet descriptor node is 32 bits (described later). The Sub-buffer Storage Space occupies 32bits×1024×24=96KB of DDR space. The Available Sub-buffer ID Pool occupies 5bits×24=120bits, or 1 BRAM.

**Figure 4:**
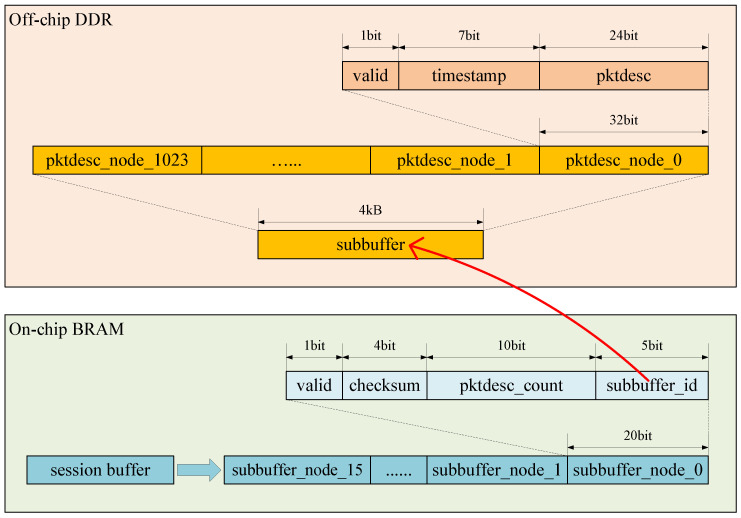
Hierarchical storage of session buffer. Sub-buffer ID serves as the address of the sub-buffer.

To achieve a deep, high-speed buffer, we employed the idea of hierarchical storage. Each session’s buffer is a 16-depth sub-buffer node array, as illustrated in Figure 4. The session sub-buffer nodes are indexed sequentially from 0 to 15. Each sub-buffer node contains a sub-buffer ID, a valid bit, a checksum, and the number of valid packet descriptor nodes in this sub-buffer. The sub-buffer node arrays with low space usage and high access frequency are stored on a chip, while the sub-buffers with large space requirements are stored in DDR. The sub-buffer ID serves as an address to access the sub-buffer in the DDR when necessary. Using a hierarchical storage strategy, we achieve a balance between on-chip resource usage and performance.

Because the width of a sub-buffer node is 20 bits, the width of a sub-buffer node array is 20bits×16=320bits. For four sessions, all the sub-buffer node arrays occupy 320bits×4=1.25Kbits, that is, 5 BRAMs.

Within a sub-buffer node, the valid bit indicates whether the related sub-buffer has been allocated (is in use) or not. If the valid bit is 1, the sub-buffer can be obtained from the DDR by the sub-buffer ID. The checksum records a random number when the sub-buffer is first allocated, which is used to verify the validity of timeout events. The number of valid packet descriptors in the sub-buffer accelerates the scanning process while handling timeout events. This will be explained in the following subsections.

Within a packet descriptor node, the valid bit indicates whether the entry in the sub-buffer has been used (cached a packet) or not. If the valid bit is 1, the packet can be retrieved from the DDR by the packet descriptor. The timestamp records the packet’s arrival time in milliseconds, which will be explained later.

### 3.4. Packet Processing

When the system receives a packet, the Scheduler extracts the session ID and packet number (PN) from the packet and forms a packet processing request, which is sent to the Session Manager for further processing.

Because each session’s buffer is a typical circular buffer consisting of multiple sub-buffers, the Index of the sub-buffer (in a sub-buffer node array) where a packet numbered PN falls can be obtained using Equation (2).

(2) Index=(PN/Subbuffer_Depth)%Array_Length.

Here, Subbuffer_depth, representing the depth of a sub-buffer containing packet descriptor nodes, is 1024, as described before. Array_Length, representing the number of elements (sub-buffers) in a sub-buffer node array, is 16. Note that “/” represents the integer division and “%” represents the modulo operation.

Additionally, the Offset of the packet descriptor node (in a sub-buffer) where a packet numbered PN is located can be calculated using Equation (3).

(3) Offset=PN%Subbuffer_Depth.

Note that “%” represents the modulo operation.

It is economic to implement Equations (2) and (3) in the hardware because both Subbuffer_Depth=1024 and Array_Length=16 are powers of two.

We maintained an Expected Packet Number (EPN) for each session, which represents the PN of the next sequential packet. The head pointer of the circular buffer points to the position of the EPN in the buffer, and it can move forward with the arrival of sequential packets or handling of timeout events. The EPN is also updated as the head pointer moves forward.

After receiving a packet processing request containing the session ID and PN, the Session Manager reads the EPN, packet timeout threshold, and sub-buffer node array of this session from BRAMs. The subsequent processes are based on the comparison results of the PN and the EPN.

If the PN is less than the EPN, the packet is regarded as a duplicate packet and is dropped. To avoid affecting retransmission, the system outputs the retransmitted packets (marked by the SD-WAN devices at the sender) for which the PN is less than the EPN.

If the PN is equal to the EPN, the packet is considered ordered and is outputted. Compared with Benevs [21], our system is improved, as we send the sequential packet out instead of storing it in the DDR. Meanwhile, if the PN falls into a valid sub-buffer, we send the consecutive packet descriptors from the position of EPN in the sub-buffer, to the Pkt Store to output ordered packets from the DDR. This process may involve multiple sub-buffers and enables the emptied sub-buffers to be freed. Due to read latency, if there are packets of the same session waiting to be retrieved from the DDR, the sequential packet has to be cached and sent by the Pkt Store instead of the Scheduler to preserve order.

If the PN is greater than the EPN, the packet may be duplicated or out of order. If the PN falls into a valid sub-buffer and the related packet descriptor node is occupied, the packet is considered a duplicate and is discarded. Otherwise, the packet is out of order. We store the out-of-order packet in the DDR and place its descriptor at the PN-related location in the sub-buffer. Sub-buffers can be allocated from the pool if needed. Whenever a sub-buffer is allocated, we initialize the corresponding sub-buffer node in the sub-buffer node array and create a timeout event for the packet. Its timeout value is the packet timeout threshold of this session.

### 3.5. Timeout Event

When a packet times out, the Timer sends a timeout event, including the session ID, sub-buffer Index, packet offset, and checksum, to the Session Manager for processing.

As discussed before, packet loss results in long-lived packet holes in session buffers, affecting packet reordering and the reuse of the memory pool. Therefore, a packet timeout mechanism is required. Since we assume that the maximum multipath delay difference is 17.5 ms in our design, the timeout value of any packet in the buffer is less than 17.5 ms and can be specified by different sessions. The Timer module is responsible for managing and triggering timeout events.

Creating a timeout event for every packet requires high performance of the Timer module. However, creating only one timeout event for the entire buffer is inaccurate, resulting in either large latency or insufficient waiting times for out-of-order packets. Hence, we use the sub-buffer as the timeout management unit. When a sub-buffer is first allocated to cache a packet, we create a timeout event for the packet. For subsequent packets falling in the same sub-buffer, we only record their arrival timestamps in the packet descriptor nodes. As a result, each session can create 16 timeout events at most, significantly reducing the Timer’s workload.

When the first packet in a sub-buffer has timed out, if we only output all the packets before the timeout packet, the sub-buffer may get rid of timeout management. However, if we clear the entire sub-buffer, it is unfair to other packets. Especially when the packet rate is low, the incoming packets in the sub-buffer are evicted without waiting for a long enough time. Therefore, we propose a timeout event handling algorithm to update the sub-buffer’s timeout value instead of emptying the sub-buffer. The algorithm (Algorithm 1) is based on packet arrival timestamps. Specifically, after receiving a timeout event, we output the packets before the timeout packet and the consecutive packets from the timeout packet. If there is an non-timeout packet after a packet hole, a new timeout event is created for the non-timeout packet. The timeout value of the created timeout event is the time remaining until the non-timeout packet expires. The algorithm keeps the packets which have not timed out in the buffer to ensure long enough waiting times for out-of-order packets in packet holes.
**Algorithm 1** Timeout Event Handling Algorithm1:Receive a timeout event. Assume the timeout packet is in sub-buffer A.2:Output all the packets before the timeout packet in the buffer;3:Output the timeout packet;4:pkt_hole_exist=0;5:**while** sub-buffer A is not empty **do**6:   Scan to the next packet descriptor node in sub-buffer A, called packet N;7:   **if** pkt_hole_exist=0 **then**8:     **if** packet N is empty **then**9:        pktdesc_hole_exist=1;10:     **else**11:        Output packet N;12:     **end if**13:   **else**14:     **if** packet N is valid and has not timed out **then**15:        Create a new timeout event for packet N;        **break**16:     **else**17:        **if** packet N is valid and has timed out **then**18:           Output packet N;19:           pktdesc_hole_exist=0;20:        **end if**21:     **end if**22:   **end if**23:**end while**

The timestamp in a packet descriptor node is used to determine whether the packet timed out or not, and calculate the new timeout value for the sub-buffer. The packet descriptor count field in a sub-buffer node is used to terminate the scanning process when the sub-buffer is empty. A randomly generated checksum is stored in the sub-buffer node and the timeout event in order to ensure the validity of the incoming timeout event, because sub-buffers are dynamically allocated and released.

## 4. Implementation and Evaluation

We developed the proposed SSPRD in Verilog and implemented it on a Sugon NetFirm-4E04 [27] FPGA board. The chip of the FPGA board is Xilinx Kintex-7 series xc7k325tffg900-2 [28]. We deployed the FPGA board on a Dell R740 [29] commodity server. There were 203,800 LUTs, 64,000 LUTRAMs, 407,600 FFs, and 445 BRAMs on the FPGA. Memory modules of 8 GB (2 × 4 GB) were installed on the FGPA board.

SSPRD was integrated into Corundum [30], and the system frequency was 200 MHz. Python-based Cocotb [31] (version 1.8.1) was used for simulation. The data width of the AXI interface of the DDR Memory Interface Generator (MIG) was 512 bits, and the data width of the AXIS interface of the packet stream was 128 bits.

### 4.1. Specifications and Resource Utilization

SSPRD achieves a sorting buffer with a maximum depth of 16,384 packets per session, which is sufficient for packet reordering and deduplication under 10 Gbps traffic with 1500-byte packets and a 17.5 ms multipath delay difference. We compared the reorder buffer depth per session with other hardware solutions in Table 1. It can be seen that SSPRD has the deepest sorting buffer, 32 times deeper than that in [16,21].

In the example implementation presented in this paper, the session count is 4 (equal to the session count in Benevs [21]) and the sub-buffer count is 24. Additionally, the architecture of SSPRD is scalable to support various session numbers or buffer depths by specifically adjusting its data structures. The storage utilization of SSPRD is shown in Table 2. After synthesizing SSPRD using Vivado 2020.2, the logical resource utilization was obtained, as shown in Table 3. With low resource and space consumption, SSPRD can easily be integrated into existing SD-WAN CPE devices.

### 4.2. Shared vs. Pre-Allocated Storage

Shared storage is superior to pre-allocated storage in terms of space occupation. During the following comparison, we assume that the bandwidth is 10 Gbps, the packet size is 1500 bytes, and the maximum multipath delay difference is 17.5 ms. Hence, the depth of a reorder buffer should be up to 15,360 packets per session. Every packet occupies 2 KB of DDR space for alignment.

**Figure 5:**
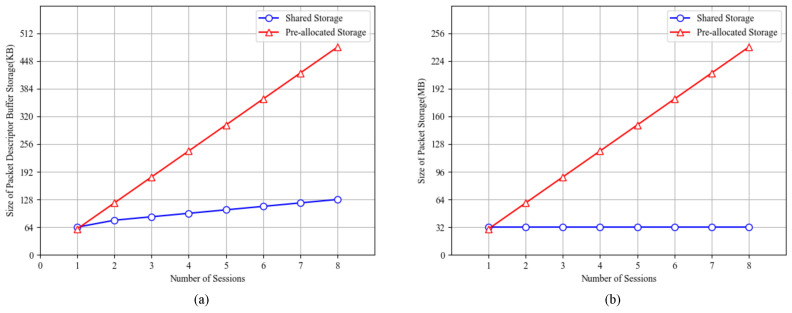
Comparison of the space usage based on shared storage strategy and pre-allocated storage strategy. (**a**) Size of packet descriptor buffer storage. (**b**) Size of packet storage.

The storage space of packets and sub-buffers in our design is shared by all sessions. Due to the shared storage strategy and the packet timeout mechanism, the storage space of packets and packet descriptor buffers is mainly determined by the network bandwidth, packet size, and maximum multipath delay difference, and is generally independent of the number of sessions. The number of sub-buffers needed is 16+2×Session_Count considering each active session requires at least two sub-buffers, as described before. Additionally if the session count is 1, the number of sub-buffers is 16 because one session can occupy 16 sub-buffers at most. As a result, we can determine the relationship between the size of buffer storage and the number of sessions, represented by a blue line in Figure 5a. The relationship between the size of packet storage and the number of sessions is represented by a blue line in Figure 5b.

However, if the packet storage space is pre-allocated (or reserved) for sessions, similarly to the design of Ukon [19], it will occupy Buffer_Depth×Pkt_Size×Session_Num of DDR space, as represented by a red line in Figure 5a. If the storage space of packet descriptor buffers is pre-allocated for sessions, similarly to the design of Benevs [21], its space consumption will be Buffer_Depth×Pktdesc_Width×Session_Num, as represented by a red line in Figure 5b. According to Figure 5, it can be concluded that the shared storage strategy dramatically reduces the storage footprint of SSPRD for storing packets and packet descriptor buffers. Compared with Benevs [21] in Figure 5a, for which the session count is also 4, SSPRD reduces the buffer space usage by 62.5%.

In Table 4, we list the packet and the buffer storage strategies of the FPGA packet reordering solutions. We can see that SSPRD first adopts the shared storage strategy for both packets and buffers. Although [16,21] employ a shared packet storage, these methods fail to determine a reasonable size of packet storage space due to the lack of packet timeout mechanism.

### 4.3. Deduplicating and Reordering Performance

**Figure 6:**
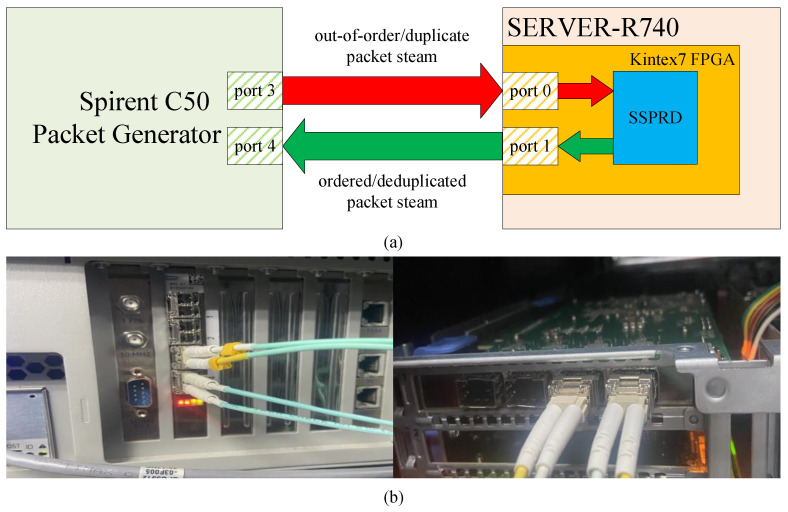
Test environment. (**a**) Test setup diagram. (**b**) Test hardware environment. Spirent C50 ports are on left side of picture, and FPGA ports are on right side.

The test environment used in this study is shown in Figure 6. Port 0 and Port 1 of the FPGA were connected to Port 3 and Port 4, respectively, of the Spirent C50 packet generator at 10 Gbps. SSPRD receives the out-of-order and duplicate packet streams from the Spirent C50, and outputs the ordered and deduplicated packet streams back to the Spirent C50 for checking. Under a specified packet rate of Spirent, the test is passed if the captured output packets from SSPRD match the expected results without any packet loss inside the FPGA.

When Spirent generates ordered (PN = EPN) or duplicate (for which PN < EPN) packets, SSPRD achieves 10 Gbps throughput because no DDR access for sub-buffers or packets is required. Next, we focus on the tests that the system needs to access the DDR.

**Figure 7:**
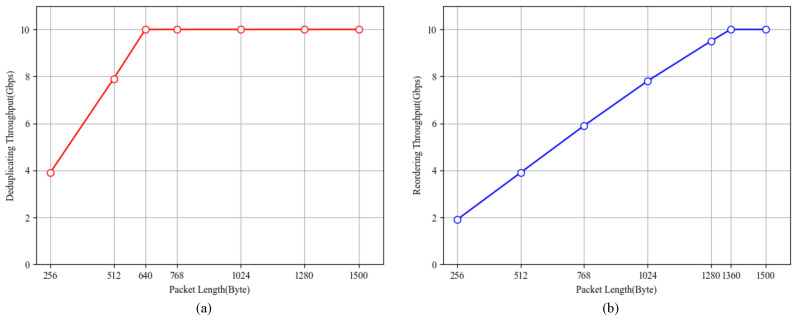
Performance test. (**a**) Deduplicating throughput. (**b**) Reordering throughput.

In the packet deduplicating test, the sequence of the PNs of the packets generated by Spirent is “x + 2, x + 2, …, x + 2”, where x is a random 32-bit number and the EPN of a session is x + 1. After the first out-of-order packet (PN = x + 2) is cached in the DDR, the subsequent packets are all duplicate packets. It is efficient to obtain a pure packet deduplicating performance. Additionally, we send a packet (PN = x + 1) last to verify that the reorder function works. Figure 7a shows the deduplicating performance of SSPRD, which reaches a throughput of 10 Gbps when the packet length is 640 bytes or greater.

In the packet reordering test, we generated an out-of-order stream with a maximum OOO distance of 15,360 as follows. Assume the EPN of a session is x + 1, where x is a random 32-bit number. First, we prepared the sequential packets from PN = x + 1 to PN = x + 15,360. Second, we exchanged the packets of PN = x + 1 and PN = x + 15,360. Third, we randomly shuffled the order of packets from PN = x + 2 to PN = x + 15,359. In this way, we created an out-of-order stream and sent it by Spirent. Figure 7b shows the reordering performance of SSPRD, which achieves 10 Gbps throughput when the packet length is 1360 bytes or greater.

We further tested SSPRD in four sessions. The packet length was set to 1500 bytes. In the four-session packet deduplicating test, Spirent sent four duplicated streams, each at a rate of 2.5 Gbps. The PN list of each stream is the same as that in the single-session duplicating test mentioned before. In the four-session packet reordering test, Spirent sent four OOO streams, each at a rate of 2.5 Gbps. The PN list of each stream is basically the same as that in the test mentioned before, except the maximum OOO distance of the PN list for each stream is 2.5Gbps×17.5ms/1500B=3646 (according to Equation (1)). In the tests, we captured the output packets from SSPRD and checked the PN list of each output stream and we verified whether there is any packet loss inside the FPGA. SSPRD passed both the packet deduplicating test and reordering test in the four sessions, with a 1500-byte packet size and a sum of 10Gbps bandwidth.

Deduplicating achieves higher throughput than sorting due to its lower DDR access frequency. Handling a duplicate packet involves reading a sub-buffer from the DDR once, while handling an out-of-order packet requires reading and writing a sub-buffer once and storing a packet in the DDR. Using High-Bandwidth Memory (HBM) instead of DDR can improve the performance of our system in the future.

Table 5 compares the reordering throughput (for 1500-byte packets) per session of the FPGA solutions, which stores out-of-order packets in off-chip memory. Hoang [16] consumes a large amount of logical resources to complete sorting in two cycles, and is unable to achieve a sorting buffer deeper than 512. Apart from the unfeasible solution, SSPRD achieves 10 Gbps reordering throughput, and outperforms the other systems. The performance of our system is sufficient for multipath transmission in WANs. Compared with Benevs [21] (system frequency is 156.25 MHz), SSPRD runs at a higher frequency of 200 MHz and Benevs stores all incoming packets in DDR. However, we checked the packet’s PN first and decided how the packet was processed later, which improved our system’s performance by reducing the number of packets written into DDR unnecessarily.

### 4.4. Handling Timeout Event

**Figure 8:**
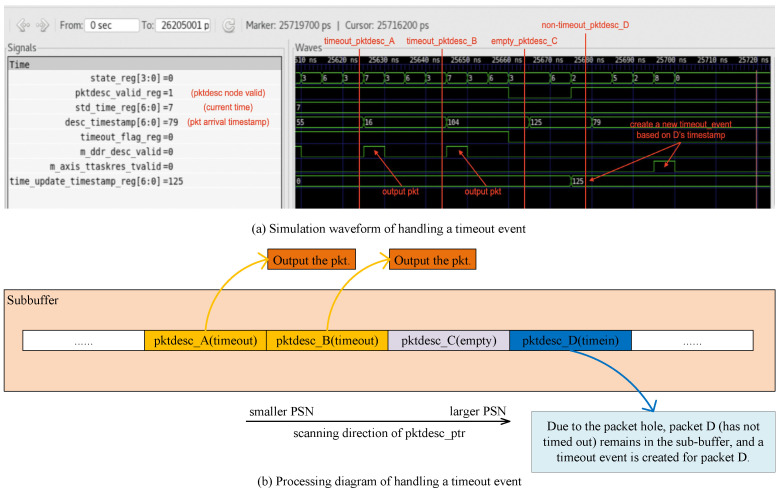
A simulation of handling a timeout event in the sub-buffer. (**a**) Simulation waveform. (**b**) Processing diagram.

Using a wave simulation, we verified that the Sub-buffer Handler module follows Algorithm 1 to handle timeout events. We use Python-based Cocotb [31] to emulate the logical behaviors of the hardware RTL (Register Transfer Level) code. Figure 8a,b show a waveform and diagram of the Sub-buffer Handler module handling a timeout event.

In the waveform, the width of the millisecond timestamp is 7 bits, cycled every 128 ms. *std_time_reg* represents the timestamp of the current time. The current timestamp is 7 ms in Figure 8a. The timestamp and valid bit of the packet descriptor nodes in the sub-buffer are *desc_timestamp* and *pktdesc_valid_reg*, respectively. In Figure 8a, the valid bits of the packet descriptor nodes A, B, C, and D are 1, 1, 0, and 1, respectively. And the timestamps of nodes A, B, and D are 55 ms, 16 ms, and 125 ms. The packet timeout threshold of this session was set to 17.5 ms. During the simulation, the module first outputs all the packets before the timeout packet, and then scans forward and outputs the consecutive packets after the timeout packet. When scanning to *pktdesc_A*, the module finds that it timed out (the current timestamp is 7 ms, packet timestamp is 55 ms, and their difference is 128−55+7=80ms>17.5ms considering the wrap-around of the 7-bit timestamp). Therefore, the module outputs packet A. The next packet descriptor node, *pktdesc_B*, also timed out (packet timestamp is 16 ms, 128−16+7=119ms>17.5ms). So the module outputs packet B. The next node, *pktdesc_C*, is empty. It is a packet hole in the sub-buffer. The next node, *pktdesc_D*, has not timed out (128−125+7=10ms<17.5ms). So, the module keeps packet D in the buffer and stops scanning forward. Meanwhile, a timeout event, for which the timeout value is 17.5ms−10ms=7.5ms, is created for packet D. The EPN of this session is updated to a PN where *pktdesc_C* is represented.

The simulation shows that, while outputting the timeout packets, our algorithm keeps the packet which has not timed out in the sub-buffer and a new timeout event is created for the sub-buffer. The timeout value of the created timeout event is the time remaining until the non-timeout packet expires.

## 5. Conclusions

In this paper, we propose SSPRD, a shared-storage-based hardware packet reordering and deduplication system for multipath transmission in WANs. The storage space of packets and buffers is shared by all sessions with dynamic allocation. We manage and sort packets in the buffer based on their descriptors instead of themselves. A sub-buffer-based timeout event handling algorithm is proposed. While supporting four sessions, SSPRD achieves deep reorder buffering on hardware, with a depth of up to 15,360 packets per session. Compared with other solutions, SSPRD reduces the buffer space usage by 62.5%, and reaches a packet reordering and deduplicating performance of 10 Gbps for 1500-byte packets.

## Figures and Tables

**Table 1:** The reorder buffer depth per session of hardware packet sorting solutions.

Design	Platform	Reorder Buffer Depth
Meitinger [18]	NP	32
Wu [15]	NP	32
Traboulsi [20]	NP	128
Zhou [17]	FPGA	43
Ukon [19]	FPGA	480
Benevs [21]	FPGA	512
Hoang [16]	FPGA	512
SSPRD	FPGA	16,384

**Table 2:** The storage utilization of SSPRD.

Data Structure	On-Chip BRAM	Off-Chip DDR
Packet Storage Space	-	32 MB
Available Packet ID Pool	224 Kbits ^1^	-
Sub-buffer Storage Space	-	80 KB
Available Sub-buffer ID Pool	120 bits ^2^	-
Session Sub-buffer Node Array	1.25 Kbits ^3^	-

^1^ Consumes 7 BRAMs. ^2^ Consumes 1 BRAM. ^3^ Consumes 5 BRAMs.

**Table 3:** The logical resource utilization of SSPRD.

Resource Type	Utilization
LUT	19,953 (9.79%)
LUTRAM	5542 (8.66%)
FF	30,366 (7.45%)
BRAM	21 (4.72%)

**Table 4:** Packet and buffer storage strategies of FPGA packet reordering solutions.

Design	Packet Storage Strategy	Buffer Storage Strategy
Zhou [17]	Pre-allocated	-
Ukon [19]	Pre-allocated	-
Hoang [16]	Shared ^1^	Consuming too many ALMs ^2^
Benevs [21]	Shared ^1^	Pre-allocated
SSPRD	Shared	Shared

^1^ Have no packet timeout mechanism. ^2^ Consume 81% ALM resource of Cyclone V FPGA under a reorder depth of 512 with 16-bit PN per session.

**Table 5:** The packet storage location and reordering throughput per session of FPGA solutions.

Design	Packet Storage Location	Throughput
Hoang [16]	Off-chip	Complete sorting in 2 cycles ^1^
Zhou [17]	Off-chip	4 Gbps
Benevs [21]	Off-chip	1 Gbps
SSPRD	Off-chip	10 Gbps

^1^ Consumes 81% of ALMs resource of Cyclone V FPGA under a reorder depth of 512 with 16-bit PN.

## Data Availability

All the necessary data are included in the article.
